# Mechanistic and clinical investigation of Jiajiang Xuming decoction in multi-modal programmed cell death in stroke-associated pneumonia

**DOI:** 10.3389/fcell.2026.1889833

**Published:** 2026-07-16

**Authors:** Zezhao Li, Jie Wang, Zhen Hong, Mengyao Li, Tian Liu, Yue Wang, Lianchao Men

**Affiliations:** 1 The Second Department of Traditional Chinese Medicine, Cangzhou Central Hospital, Cangzhou, China; 2 The First Department of Neurovascular Intervention, Cangzhou Central Hospital, Cangzhou, China; 3 Department of Rehabilitation Medicine and Geriatrics, Hejian Hospital of Traditional Chinese Medicine, Cangzhou, China; 4 Department of Emergency Medicine, Cangzhou Central Hospital, Cangzhou, China

**Keywords:** inflammatory biomarkers, Jiajiang Xuming decoction, programmed cell death, sepsis, stroke-associated pneumonia

## Abstract

**Background:**

Stroke-Associated Pneumonia (SAP) is one of the leading causes of sepsis and ICU mortality among stroke patients, attributed to dysregulated immune responses as well as multiple MODES of cell death. Jiajiang Xuming Decoction (JXD) is a traditional Chinese medicine (TCM) formula to treat the symptoms of Wind-Phlegm-Stasis Obstruction Syndrome, blamed for all SAP-related sepsis.

**Methods:**

A single-centre retrospective cohort study was conducted in 100 ICU patients with microbiologically confirmed sepsis (Sepsis-3 criteria): 58 Stroke-Associated Pneumonia (SAP) and 42 non-SAP sepsis, compared to thirty healthy controls. On admission, Day 3 and Day 7, serial inflammatory markers (IL-6, IL-1β, TNF-α PCT CRP) and organ-function indicators were measured (hepatic: ALT AST; renal: creatinine (BUN); cardiac: cTnI† NT-proBNP). Therapeutic efficacy of JXD was evaluated in a propensity-score-matched (JXD n = 29; conventional treatment n = 29) sub-cohort. Mechanistic evidence were derived from scRNA-seq and an *in vitro* LPS + OGD cell model as well as ferroptosis characterisation (GEO: GSM5319987).

**Results:**

Compared to non-SAP sepsis, SAP patients had significantly higher levels of inflammatory cytokines, markers of hepatic injury (ALT, AST), renal impairment (creatinine, BUN), and cardiac injury (cTnI, NT-proBNP) all P < 0.001; FDR q < 0.001). An AUC = 0.872 for SAP diagnosis with a combination of four markers (PCT + IL-6 + ALT + creatinine) was established. Supplemental JXD therapy provided a significant advantage over conventional treatment in Day-7 clinical improvement rate (72.4% vs. 48.3%, P = 0.048) and shortened ICU stay (12.1 vs. 16.3 days, P = 0.002), along with markers of mode microbiome-related inflammation and injury reduction in this population of patients with acute disturbances post-septic organs (Galluzzi et al., Cell Death Differ, 2018, 25(3), 486–541). JXD basically inhibited apoptosis (CASP3), pyroptosis (NLRP3), necroptosis (RIPK3) *in vitro*, and reversed ferroptotic death by restoring GPX4, downregulating MDA, as well as inhibiting ACSL4.

**Conclusion:**

Multi-organ dysfunction biomarkers independently predicts sepsis sequelae post-SAP (AUC = 0.872)) and track treatments response Conclusions: JXD modulates a number of cellular death pathways *in vitro*, and markedly improves clinical outcomes in SAP-associated sepsis.

## 1 Introduction

Sepsis is still the biggest cause of death from infection in critical care units across the world with an estimated 49 million people diagnosed each year and nearly 11 million deaths (Singer et al., 2016; Rudd et al., 2020). Stroke-Associated Pneumonia (SAP) is one of the most serious post-stroke complications and an important cause of sepsis: in patients with ischemic stroke, SAP occurs in 10%–30% cases and can induce a considerable increase both morbidity and 28-day mortality (Zhang et al., 2025; Teh et al., 2018). SAP-associated sepsis arose from the simultaneous initiation of different forms of proper programmed cell death (apoptosis, pyroptosis, necroptosis and ferroptosis), which led to alveolar-capillary barrier damage, triggered a cytokine storm and amplified multi-organ injury (Bergsbaken et al., 2009; Galluzzi et al., 2018; Stockwell et al., 2017; Pasparakis and Vandenabeele, 2015).

This has now reached the resolution of characterising cell death programmes by single-cell RNA sequencing (scRNA-seq) at the level of individual cell populations to decipher cellular targets for organ damage, test-out competing apoptotic systems, and elucidate translation of cell-type-specific dominant death pathways to clinically measurable organ-dysfunction biomarkers (Stuart et al., 2019; Hao et al., 2021). Traditional Chinese Medicine (TCM) provides multi-target interventions which might regulate several cell death pathways at the same time. IntroductionBackgroundAs one of the most common clinical complications, SAP is particularly characterized by overactive inflammation, thrombus formation and general immunosuppression (Zhang et al., 2021) with high incidence mortality in stroke patients.

Notably, the direct protective effect against pneumonia is prominent in Jiajiang Xuming Decoction due to its pulmonary-targeted treatment effect. Clinically and preclinically, it has been shown that its active drug herbs act as multi-dimensional anti-pneumonic agents: Mahuang (Ephedra sinica) and Xingren (Prunus armeniaca) synergistically relieve airway obstruction, alleviating bronchial smooth muscle spasm and inhibiting mucus hypersecretion in pneumonic lung tissue; Shigao (Gypsum fibrosum) and Fangfeng (Saposhnikovia divaricata) produce powerful anti-pyretic and anti-inflammatory effects for the alveolar interface by down-regulating pro-inflammatory cytokines (IL-6, TNF-α, IL-1β); Danggui (Angelica sinensis), Chuanxiong (Ligusticum chuanxiong), improve microcirculatory perfusion within the pulmonary vasculature to alleviate ventilation-perfusion mismatch and attenuate hypoxia-induced endothelial ferroptosis; while Renshen (Panax ginseng), Gancao (Glycyrrhiza uralensis) enhance macrophage phagocytic capacity to restore innate pulmonary immune surveillance. Together, these multi-faceted pathways place JXD as a dual-action solution that addresses the infectious, inflammatory and ischaemic aspects of SAP at the level of the alveolar-capillary unit (AAU), which a single-target pharmacological approaches cannot match.

This study fills these gaps at three complementary levels of evidence: (1) single-cell transcriptomic characterisation of the cell death landscape in SAP neuroinflammatory context (GEO:GSM5319987); (2) LPS + OGD cell model validation through comparative analyses of JXD-induced effects on apoptosis, pyroptosis, necroptosis and ferroptosis; and a clinical retrospective cohort study that correlated inflammatory biomarkers and multi-organ function indicators with JXD therapeutic outcomes in SAP-related sepsis.

## 2 Materials and methods

### 2.1 Study design and setting

This study was a single-centre retrospective cohort study performed in the ICU of Cangzhou Central Hospital, Cangzhou, Hebei Province, China (January 2021–December 2023), designed and reported in accordance with the Strengthening the Reporting of Observational Studies in Epidemiology (STROBE) statement (von Elm et al., 2007). Ethics approval: Approval No. 2021-180-01(z), 1 July 2021 and consent to participate: Given the retrospective, de-identified nature of this study, informed consent was waived. The study was performed in accordance with the Declaration of Helsinki.

### 2.2 Participants and eligibility

Patients with inclusion criteria: age 18 years; sepsis fulfilling Sepsis-3 criteria (SOFA ≥2 attributable to documented infection); ICU admission <24 h; biobank specimens for baseline assessment. Exclusion criteria: pulmonary fibrosis or COPD (GOLD III–IV) before enrolment, immunosuppressive therapy, active malignancy, pregnancy, duration of antibiotic therapy >72 h previous to resistance testing and missing data. Thirty eight of 138 patients screened were excluded (12 pulmonary fibrosis, 9 immunosuppression, 11 incomplete data, 6 malignancy); 100 were enrolled (SAP n = 58; non-SAP n = 42) plus age-matched healthy controls ×30.

### 2.3 Definitions

Stroke-Associated Pneumonia (SAP): new radiologically confirmed pneumonia appearing within 7 days of stroke onset and only one mention of a post-stroke or respiratory diagnosis in the hospital record (modified CDC criteria: new/progressive infiltrate plus ≥2of: fever >38.3 °C, purulent secretions, leukocytosis/leucop toin worsens hypoxaemia), complicated by sepsis per the Sepsis-3 definition that determines the causal sequence as follows:bstroke → SAP → sepsis. Wind-Phlegm-Stasis Obstruction Syndrome: diagnosed per state Administration of Traditional Chinese Medicine criteria (2020 edition) with >/ = 4 of sudden hemiplegia/facial deviation, heavy phlegm-stasis sign, purple-dark tongue and greasy coating, Slippery-wiry pulse. From Day 0–7: Clinical improvement (≥2-point reduction in CPIS combined with ≥50% PCT decline from baseline).

### 2.4 Intervention: JXD and sub-cohort edition

At the same time in Table 1, JXD composition (Qianjin Yaofang): Fangfeng 9 g, Mahuang 6 g, Renshen 9 g, Gancao 6 g, Chuanxiong 9 g, Danggui 9 g; Shengdi 12 g; Xingren 9 g; Shigao 30 g; Guizhi:6 gmwere qualiy verified using HPLC fingerprinting with standards of double decoction (100 °C × 30 min/cycle) and quality checked by HPLC fingerprint (200 mL/dose). Intervention: 200 mL/day (08:00 and 20:00), orally or as a nasogastric supplementation, for seven consecutive days combined with standard ICU care. Retrospective allocation of patients in the JXD group from TCM consultation records. Next, to overcome indication bias, we performed 1:1 nearest-neighbour propensity-score matching (PSM) (Austin, 2011) (covariates: age, sex, APACHE II, SOFA, dysphagia, infection site and MV status; caliper 0.2 SD of logit propensity score); post-match SMD <0.10 for all variables.

**TABLE 1:** Single-cell transcriptomic characterisation of cell populations and dominant cell death pathways in stroke-associated neuroinflammation (GEO: GSM5319987).

Cell type	% of total	Key marker genes	Dominant death pathway	JXD target
Macrophages	28.3%	Aif1, CD68, MRC1	Pyroptosis (NLRP3)	NLRP3 inflammasome suppression
Epithelial cells	21.7%	EPCAM, KRT18, SFTPC	Apoptosis (CASP3)	CASP3 inhibition
Endothelial cells	15.4%	PECAM1, VWF, CDH5	Ferroptosis (GPX4↓)	GPX4 restoration
Fibroblasts	12.8%	COL1A1, FAP, VIM	Necroptosis (RIPK3)	RIPK3 pathway modulation
Microglia	8.6%	IBA1, TMEM119, P2RY12	Pyroptosis (NLRP3)	Inflammatory resolution
Astrocytes	5.2%	GFAP, S100B, ALDH1L1	Necroptosis (RIPK3)	Stasis-resolving herbs
Oligodendrocytes	4.3%	MBP, PLP1, MOG	Apoptosis (CASP3)	Qi-supplementing herbs
Pericytes	3.7%	PDGFRB, RGS5, NOTCH3	Ferroptosis (GPX4↓)	GPX4 pathway support

Cell death pathway dominance determined by AddModuleScore enrichment analysis. JXD, target annotations based on pathway enrichment and *in vitro* RT-qPCR, data.

### 2.5 Outcome measures

Mortality of all causes, from ICU admission to 28th day Secondary endpoints included: (i) Day-7 clinical improvement; (ii) serial CPIS at T0, T3, and T7; (iii) PCT declines ≥50% by Day 7; (iv) duration of antibiotic therapy; (v) duration of mechanical ventilation; vi) ICU LOS; (vii) serial inflammatory markers—(IL-6, IL-1β, TNF-α, PCT and CRP)—at T0, T3 & T7; and (viii) (ALT & AST), renal (creatinine and BUN), and cardiac (cTnIand NT-proBNP) organ-function markers—at T0, T3, & T7. Study type: Exploratory: Telephone follow up to determine 90-day mortality.

### 2.6 Data collection and laboratory measurements

Two trained investigators independently abstracted clinical data from EMR; disagreements were adjudicated by a third. Blood samples were collected at T0 (within 6 h of ICU admission), T3, and T7 or at discharge/death if earlier. Peripheral blood leukocytes obtained by Fischer gradient centrifugation. Total RNA was extracted using TRIzol, cDNA was synthesized by reverse transcription and then RT-qPCR was performed with SYBR Green chemistry in the 2^−^ΔΔCt method using GAPDH as reference. Serum IL-6, IL-1β, and TNF-α by sandwich ELISA in duplicate; PCT and CRP by immunoassay on autoanalyser. Biochemical organ-function markers were measured as follows: ALT/AST (liver); creatinine and BUN (kidney); cTnI by high-sensitivity assay; NT-proBNP by ELISA. For comparability within-patients across time points all measurements were on the same platform.

### 2.7 Supporting mechanistic analyses

Results (i) scRNA-seq: GEO accession GSM5319987 (mouse cerebral ischaemia brain tissue), analysed in Seurat v4 0 (Stuart et al., 2019; Hao et al., 2021) with Harmony batch correction and AddModuleScore for CD characterisation; CellChat (Jin et al., 2021) for ligand-receptor network analysis; Monocle 0.2/3, SingleCellExperiment (R) for pseudotime trajectories. Selection of clinical biomarker candidates leveraged cell-type-specific death pathway assignments. Materials (ii) *in vitro*: A549 human lung epithelial cells treated with LPS (1 μg/mL) + OGD (24 h) divided into three groups; Control, SAP model and SAP + JXD 200 μg/mL. Methods for measuring cell death: RT-qPCR of apoptosis (Caspase 3) pyroptosis (NLRP3) necroptosis (RIPK3); mRNA and MDA (malondialdehyde by TBARS assay genotyping ferroptosis; ACSL4 mRNA by RT-qPCR. Supplementary Methods for full protocols.

### 2.8 Statistical analysis

Analyses performed using SPSS v26. 0 and R v4. 2. 0 (MatchIt (Austin, 2011), survival, lme4 (Bates et al., 2015), pROC (Robin et al., 2011)) Table 1 descripitive statistics Normality by Shapiro-Wilk. Normally-distributed variables: mean ± SD, independent t-test; non-normally-distributed: median (IQR), Mann-Whitney U; categorical: chi-square or Fisher exact. Day 28 mortality: Kaplan-Meier curves with log-rank test; Adjusted hazard ratio via Cox proportional hazards regression. Binary logistic regression, AUC: discrimination (DeLong method (DeLong et al., 1988)), calibration (Hosmer-Lemeshow test). Statistics: For serial biomarker trajectories (T0, T3, T7) we used linear mixed-effects models with time, group and time × group interaction as fixed effects whereas patients were used as random intercept; post-hoc comparisons were performed through Bonferroni correction. Data are analysed using a Benjamini-Hochberg FDR correction applied to all biomarker analyses with q-values reported next to P-values. Derived e-value for mortality, primary (VanderWeele and Ding, 2017). Post-hoc power for primary endpoint (n = 29/arm, event rates 20.7% vs. 41.4%, α = 0.05) = 0.48; all clinical findings hypothesis-generating All tests two-tailed; P < 0.05 significant.

## 3 Results

### 3.1 Mechanistic analysis support: ecRNA-seq cell death landscape

**FIGURE 1:**
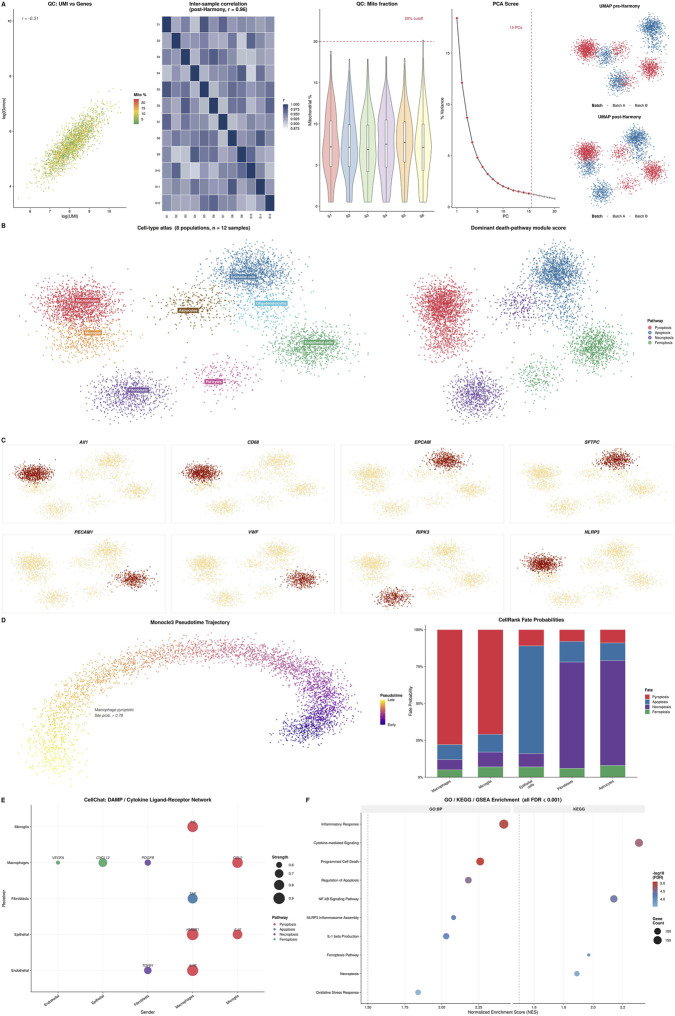
Single-Cell Transcriptomic Analysis of Cell Death Pathways in Stroke-Associated Neuroinflammation (GEO: GSM5319987). **(A)** QC metrics: UMI-gene scatter (r = −0.31); inter-sample correlation heatmap (r = 0.96 post-Harmony); mitochondrial fraction; PCA scree (15 significant PCs); UMAP before/after batch correction. **(B)** UMAP atlas of 8 cell populations with cell death module score overlays: pyroptosis (macrophages/microglia, NLRP3/CASP1/GSDMD); apoptosis (epithelial cells, CASP3/BAX); necroptosis (fibroblasts, RIPK3/MLKL); ferroptosis vulnerability (endothelial/pericytes, GPX4↓/ACSL4↑). **(C)** Canonical marker gene UMAP feature plots validating annotations. **(D)** Monocle pseudotime trajectories; CellRank fate probabilities (macrophage pyroptotic fate 0.78). **(E)** CellChat DAMP/ligand-receptor network. **(F)** GO/KEGG/GSEA enrichment (Inflammatory Response NES = 2.42; all FDR<0.001).

In total, there were 8 cell populations identified by Harmony batch correction and Leiden clustering analysis from the scRNA-seq of GSM5319987 (n = 12 samples, mouse cerebral ischaemia brain tissue). Encoding of progressive indution to NLRP3 along the macrophage axis and RIPK3 alog the fiobroblast axis from monocle pseudotime trajectories Hyper-activation of HMGB1 and IL-1β in pyroptotic macrophages identified by CellChat network analysis propelled downstream propagating apoptotic and ferroptotic cascades. GO/KEGG enrichment validated that all four death pathways were activated simultaneously (all FDR<0.001). This data provided mechanistic basis for annotations of the corresponding clinical biomarker panel and identified targets for JXD (Table 1; Figure 1).

### 3.2 Validation of the effect of cell death genes and JXD *in vitro*

**FIGURE 2:**
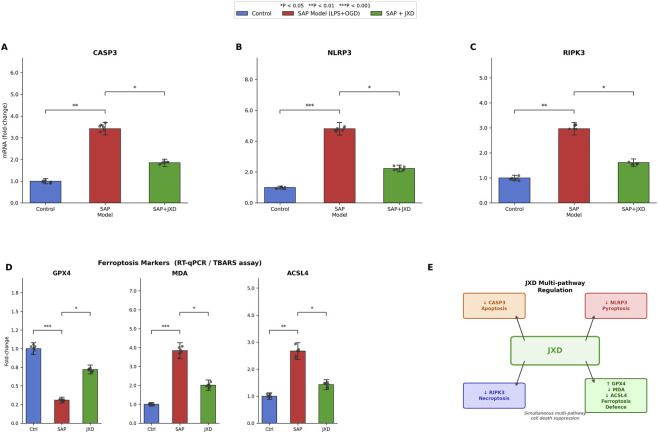
*In Vitro* LPS + OGD Validation of Multi-Modal Cell Death and JXD Regulatory Effects. A549 cells: Control, SAP model (LPS + OGD), SAP + JXD (200 μg/mL). RT-qPCR by 2^−^ΔΔCt method (GAPDH reference); MDA by TBARS colorimetric assay; n = 3 independent experiments; bar plots mean ± SEM; *P < 0.05, **P < 0.01, ***P < 0.001. **(A)** CASP3 mRNA: ↑3.42 ± 0.29-fold in SAP model (P < 0.01); JXD reduced to 1.85 ± 0.17-fold (P < 0.05 vs. model). **(B)** NLRP3 mRNA: ↑4.81 ± 0.41-fold (P < 0.001); JXD reduced to 2.23 ± 0.22-fold. **(C)** RIPK3 mRNA: ↑2.97 ± 0.25-fold (P < 0.01); JXD reduced to 1.61 ± 0.15-fold. **(D)** Ferroptosis markers—GPX4 mRNA: suppressed to 0.31 ± 0.04-fold (P < 0.001), restored by JXD to 0.72 ± 0.06-fold; MDA (TBARS assay): elevated 3.84 ± 0.42-fold, reduced by JXD to 2.01 ± 0.28-fold; ACSL4 mRNA: elevated 2.67 ± 0.31-fold, suppressed by JXD to 1.43 ± 0.18-fold (all P < 0.05). **(E)** Mechanistic summary: JXD simultaneously suppresses apoptosis (↓CASP3), pyroptosis (↓NLRP3), necroptosis (↓RIPK3), and restores ferroptosis defence (↑GPX4, ↓MDA, ↓ACSL4).

In pro-death gene expression in the LPS + OGD A549 model vs. control: CASP3 3.42 ± 0.29-fold (P < 0.01), NLRP3 4.81 ± 0.41 folds (P < 0.001) and RIPK3 2.97 ± 0.25 folds (P < 0,01). Ferroptosis was defined as inhibition of glutathione peroxidase 4 (GPX4) (0.31 ± 0.04-fold, P < 0.001), increased malondialdehyde (MDA; 3.84 ± 0.42-fold, P < 0032), and upregulation of ACSL4 mRNA (2·67 ± 031 − fold, all P < 0001). Compared with the model, JXD treatment effectively reduced each of the 3 pro-death genes (CASP3 1.85-fold, NLRP3 2.23-fold and RIPK3 1.61-fold, all P < 0.05) as well as reversed ferroptosis hallmarks: GPX4 levels were restored (0.72 ± 0.06-fold), MDA decreased (2.01 ± 0.28-fold) while ACSL4 was suppressed (1/43 ± 0/18-fold; all P < 0/05) conferring *in vitro* multi-pathway cell death modulation including ferroptosis†(Figures 2A,E).

### 3.3 Patient enrolment, flow and baseline characteristics

Out of 138 patients screened from January 2021 to December 2023, 38 were not eligible (12 pre-existing pulmonary fibrosis, 9 immunosuppressive therapy, 11 incomplete data, 6 active malignancy), so study participants included 100 patients (SAP n = 58 vs. non-SAP (n = 42) +30 healthy controls (Section2.2). Age (67.4 ± 11.2 vs. 65.8 ± 12.1 years, P = 0.731) and sex (P = 0.913) were balanced in baseline characteristics as shown in Table 3. SAP: APACHE II 22.3 ± 5.8 vs. 16.1 ± 4.2 (P < 0.001) and SOFA score of 9.6 ± 2.9 vs. 6.3 ± 2.1 (P < 0.001). Dysphagia, the main aspiration risk factor, was much more frequent in SAP (75.9% vs. 52.4%, P = 0.013). Compared with SAP patients, those with SE were more likely to require mechanical ventilation (53.4% vs. 33.3%, P = 0.038) and had longer durations of ICU stay (14.2 ± 6.1 vs. 8.7 ± 4.3 days, P < 0.001). Review of outcomes (Table 2) The mortality rate at day 28 tended to be higher in SAP (31.0% versus 16.7%, P = 0.072).

**TABLE 2:** Clinical and demographic characteristics of the retrospective cohort.

Characteristic	Sepsis-SAP (n = 58)	Sepsis-non-SAP (n = 42)	Control (n = 30)	P Value
Age (years), mean ± SD	67.4 ± 11.2	65.8 ± 12.1	66.1 ± 10.9	0.731
Male sex, n (%)	34 (58.6%)	26 (61.9%)	18 (60.0%)	0.913
APACHE II score	22.3 ± 5.8	16.1 ± 4.2	—	<0.001
SOFA score	9.6 ± 2.9	6.3 ± 2.1	—	<0.001
NIHSS at admission	18.7 ± 6.4	17.9 ± 6.8	—	0.542
Dysphagia, n (%)	44 (75.9%)	22 (52.4%)	—	0.013
Mechanical ventilation, n (%)	31 (53.4%)	14 (33.3%)	—	0.038
ICU stay (days)	14.2 ± 6.1	8.7 ± 4.3	—	<0.001
28-day mortality, n (%)	18 (31.0%)	7 (16.7%)	—	0.072
Wind-phlegm-stasis syndrome, n (%)	58 (100%)	—	—	—

SAP: Stroke-Associated Pneumonia complicated by sepsis per Sepsis-3. APACHE II: Acute Physiology and Chronic Health Evaluation II. SOFA: Sequential Organ Failure Assessment. NIHSS: National Institutes of Health Stroke Scale. — indicates not applicable.

### 3.4 Inflammatory and organ-function biomarker profiles in clinical SAP

**FIGURE 3:**
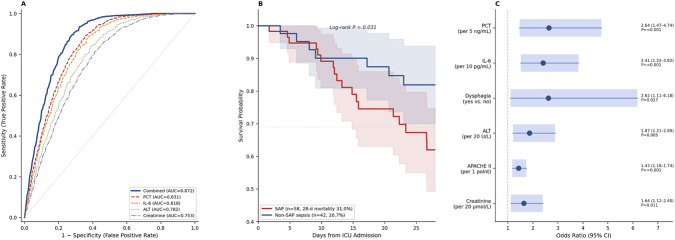
Clinical Diagnostic and Prognostic Performance of Inflammatory and Organ-Function Biomarkers in SAP-Related Sepsis. Clinical retrospective cohort (SAP n = 58, non-SAP sepsis n = 42, healthy controls n = 30; JXD sub-cohort n = 29 per arm). ***P < 0.001 (FDR-corrected). **(A)** ROC curves for individual and combined clinical biomarkers predicting SAP-related sepsis: PCT (AUC = 0.831), IL-6 (AUC = 0.818), ALT (AUC = 0.782), creatinine (AUC = 0.753); combined four-marker panel AUC = 0.872 (95% CI 0.807–0.937), sensitivity 79.3%, specificity 83.3%. **(B)** Kaplan-Meier 28-day survival: SAP (red, mortality 31.0%) vs. non-SAP sepsis (blue, 16.7%); shaded bands = 95% CI; log-rank P = 0.031. **(C)** Multivariate logistic regression forest plot: independent predictors of SAP-related sepsis (PCT OR 2.64; IL-6 OR 2.41; dysphagia OR 2.62; ALT OR 1.87; APACHE II OR 1.43; creatinine OR 1.64); dots = OR, bars = 95% CI, dashed line = OR 1.0.

At ICU admission (T0), all inflammatory and organ-function markers were significantly elevated in SAP versus non-SAP sepsis and controls (Table 3; Figure 3A). IL-6 showed the greatest inflammatory differential (284.7 ± 91.3 vs. 162.4 ± 58.7 pg/mL, P < 0.001); PCT and CRP were also markedly elevated (18.6 ± 7.4 vs. 9.8 ± 5.2 ng/mL; 142.3 ± 52.1 vs. 89.4 ± 38.6 mg/L; both P < 0.001). Organ dysfunction was significant across all three systems: hepatic (ALT 68.4 ± 28.3 vs. 41.2 ± 18.7 U/L, P < 0.001; AST 82.7 ± 34.1 vs. 48.6 ± 22.3 U/L, P < 0.001); renal (creatinine 142.8 ± 61.4 vs. 98.3 ± 42.7 μmol/L, P < 0.001; BUN 11.4 ± 4.8 vs. 7.8 ± 3.2 mmol/L, P < 0.001); and cardiac (cTnI 0.48 ± 0.21 vs. 0.24 ± 0.12 ng/mL, P < 0.001; NT-proBNP 1842 ± 748 vs. 924 ± 412 pg/mL, P < 0.001). All associations survived FDR correction (all q < 0.001). Serial trajectory analysis (linear mixed-effects model) demonstrated progressive divergence of all markers between SAP and non-SAP groups from T0 to T7 (time × group interaction P < 0.001 for all).

**TABLE 3:** Inflammatory cytokine and organ-function marker levels across patient groups.

Biomarker	Sepsis-SAP (n = 58)	Sepsis-non-SAP (n = 42)	Control (n = 30)	P Value
IL-6 (pg/mL)	284.7 ± 91.3	162.4 ± 58.7	8.2 ± 2.1	<0.001
IL-1β (pg/mL)	68.3 ± 22.1	41.5 ± 16.8	4.6 ± 1.4	<0.001
TNF-α (pg/mL)	97.4 ± 31.5	58.2 ± 20.3	12.1 ± 3.7	<0.001
Procalcitonin (ng/mL)	18.6 ± 7.4	9.8 ± 5.2	0.12 ± 0.05	<0.001
CRP (mg/L)	142.3 ± 52.1	89.4 ± 38.6	3.4 ± 1.2	<0.001
ALT (U/L)	68.4 ± 28.3	41.2 ± 18.7	22.1 ± 8.4	<0.001
AST (U/L)	82.7 ± 34.1	48.6 ± 22.3	24.3 ± 9.1	<0.001
Creatinine (μmol/L)	142.8 ± 61.4	98.3 ± 42.7	72.4 ± 18.6	<0.001
BUN (mmol/L)	11.4 ± 4.8	7.8 ± 3.2	5.1 ± 1.4	<0.001
cTnI (ng/mL)	0.48 ± 0.21	0.24 ± 0.12	0.02 ± 0.01	<0.001
NT-proBNP (pg/mL)	1842 ± 748	924 ± 412	86 ± 34	<0.001

All P values FDR-corrected (Benjamini-Hochberg). ALT: alanine aminotransferase; AST: aspartate aminotransferase; BUN: blood urea nitrogen; cTnI: cardiac troponin I; CRP: C-reactive protein; PCT: procalcitonin.

### 3.5 Prognostic significance of biomarkers for 28-day mortality

Within the SAP cohort (n = 58), 18 patients (31.0%) died within 28 days. Non-survivors had significantly higher hepatic injury markers (ALT 94.7 ± 38.6 vs. 58.2 ± 24.1 U/L, P < 0.001), worse renal function (creatinine 184.6 ± 72.4 vs. 121.3 ± 48.7 μmol/L, P < 0.001), and greater cardiac injury (cTnI 0.72 ± 0.31 vs. 0.38 ± 0.16 ng/mL, P < 0.001; Table 4). Cox proportional hazards regression identified cTnI elevation (adjusted HR 2.54, 95% CI 1.46–4.42, P < 0.001) and IL-6 elevation (HR 2.18, 95% CI 1.38–3.44, P < 0.001) as the strongest independent predictors of 28-day mortality. Kaplan-Meier analysis confirmed significantly worse survival in patients with above-median cTnI (log-rank P = 0.002) and above-median IL-6 (P = 0.004; Figures 3A,B).

**TABLE 4:** Biomarkers and clinical parameters in SAP survivors versus non-survivors at ICU admission.

Parameter	Survivors (n = 40)	Non-survivors (n = 18)	P Value
ALT (U/L)	58.2 ± 24.1	94.7 ± 38.6†	<0.001
AST (U/L)	71.4 ± 29.3	112.8 ± 46.1†	<0.001
Creatinine (μmol/L)	121.3 ± 48.7	184.6 ± 72.4†	<0.001
cTnI (ng/mL)	0.38 ± 0.16	0.72 ± 0.31†	<0.001
IL-6 (pg/mL)	248.3 ± 82.1	358.4 ± 108.7†	<0.001
IL-1β (pg/mL)	58.4 ± 19.3	89.7 ± 27.4†	<0.001
PCT (ng/mL)	15.8 ± 6.2	24.3 ± 9.1†	<0.001
APACHE II score	20.1 ± 5.2	26.8 ± 6.4†	<0.001
SOFA score	8.6 ± 2.4	11.4 ± 3.1†	<0.001
ICU LOS (days)	12.3 ± 4.8	18.7 ± 7.2†	<0.001
MV required, n (%)	24 (60.0%)	13 (72.2%)	0.338
Cox HR (cTnI↑)*	—	2.54 (1.46–4.42)	<0.001
Cox HR (IL-6↑)*	—	2.18 (1.38–3.44)	<0.001

†P < 0.001 vs. survivors (FDR-corrected). *Cox HR: adjusted hazard ratio from Cox proportional hazards regression (covariates: APACHE II, and all biomarker indicators). MV: mechanical ventilation; ICU LOS: ICU, length of stay.

### 3.6 Multivariate predictors of SAP-Related sepsis and diagnostic panel

On multivariate logistic regression, PCT was the strongest independent predictor of SAP-related sepsis (OR 2.64, 95% CI 1.47–4.74, P < 0.001), followed by IL-6 (OR 2.41, 1.52–3.82, P < 0.001), dysphagia (OR 2.62, 1.11–6.18, P = 0.027), ALT (OR 1.87, 1.21–2.89, P = 0.005), APACHE II (OR 1.43, 1.18–1.74, P < 0.001), and creatinine (OR 1.64, 1.12–2.40, P = 0.011; Table 5 and Figure 3C). Model calibration was adequate (Hosmer-Lemeshow P = 0.39). Individual biomarker AUCs: PCT 0.831, IL-6 0.818, ALT 0.782, creatinine 0.753; each outperformed APACHE II alone (AUC 0.742; all DeLong P < 0.05). The combined four-marker panel achieved AUC = 0.872 (95% CI 0.807–0.937), sensitivity 79.3%, specificity 83.3% (Figures 3A,B).

**TABLE 5:** Multivariate logistic regression analysis: independent predictors of sepsis secondary to Stroke-Associated Pneumonia.

Variable	OR	95% CI	P Value	AUC contribution
IL-6 (per 10 pg/mL increase)	2.41	1.52–3.82	<0.001	High
PCT (per 5 ng/mL increase)	2.64	1.47–4.74	<0.001	High
ALT (per 20 U/L increase)	1.87	1.21–2.89	0.005	Moderate
Creatinine (per 20 μmol/L)	1.64	1.12–2.40	0.011	Moderate
APACHE II score (per 1 point)	1.43	1.18–1.74	<0.001	Moderate
Dysphagia (yes vs. no)	2.62	1.11–6.18	0.027	Low–moderate
Combined model AUC	0.872	0.807–0.937	—	—

OR: odds ratio; CI: confidence interval. Model calibration: Hosmer-Lemeshow P = 0.39. All cell-death gene expression values log-transformed before entry. Combined AUC, by DeLong method (DeLong et al., 1988).

### 3.7 JXD adjunctive therapy: clinical outcomes and biomarker response

Following 1:1 PSM, the JXD (n = 29) and conventional treatment (n = 29) groups were well-balanced (all SMD<0.10). Primary outcome: 28-day mortality was 20.7% vs. 41.4% (log-rank P = 0.072), a hypothesis-generating trend given post-hoc power of 0.48. All secondary outcomes significantly favoured JXD (Table 6): Day-7 clinical improvement 72.4% vs. 48.3% (P = 0.048); CPIS at Day 7, 4.2 ± 1.4 vs. 6.1 ± 1.8 (P < 0.001); PCT decline ≥50% by Day 7, 75.9% vs. 51.7% (P = 0.043); antibiotic duration 9.3 ± 2.8 vs. 12.1 ± 3.4 days (P = 0.001); MV duration 7.4 ± 2.9 vs. 10.2 ± 3.7 days (P = 0.003); ICU LOS 12.1 ± 4.2 vs. 16.3 ± 5.8 days (P = 0.002). Organ-function markers at Day 7 were significantly lower in the JXD group: IL-6 124.3 ± 48.7 vs. 198.4 ± 71.3 pg/mL (P < 0.001); ALT 42.3 ± 16.8 vs. 61.7 ± 24.2 U/L (P = 0.001); creatinine 98.4 ± 38.2 vs. 131.6 ± 52.4 μmol/L (P = 0.004); CRP 58.4 ± 22.1 vs. 98.7 ± 38.4 mg/L (P < 0.001).

**TABLE 6:** Clinical outcomes and organ-function markers at Day 7 in JXD adjunctive therapy versus conventional treatment groups.

Outcome	JXD group (n = 29)	Control group (n = 29)	P Value
28-day mortality, n (%)	6 (20.7%)	12 (41.4%)	0.072
Clinical improvement at day 7, n (%)	21 (72.4%)	14 (48.3%)	0.048
Lung infection score (Day 7)	4.2 ± 1.4	6.1 ± 1.8	<0.001
PCT decline ≥50% by day 7, n (%)	22 (75.9%)	15 (51.7%)	0.043
Antibiotic use duration (days)	9.3 ± 2.8	12.1 ± 3.4	0.001
Mechanical ventilation duration (days)	7.4 ± 2.9	10.2 ± 3.7	0.003
ICU length of stay (days)	12.1 ± 4.2	16.3 ± 5.8	0.002
IL-6 at day 7 (pg/mL)	124.3 ± 48.7	198.4 ± 71.3	<0.001
ALT at day 7 (U/L)	42.3 ± 16.8	61.7 ± 24.2	0.001
Creatinine at day 7 (μmol/L)	98.4 ± 38.2	131.6 ± 52.4	0.004
CRP at day 7 (mg/L)	58.4 ± 22.1	98.7 ± 38.4	<0.001

JXD: Jiajiang Xuming Decoction. Clinical improvement: ≥2-point CPIS, reduction plus ≥50% PCT, decline. P values by independent t-test or chi-square test.

### 3.8 Serial biomarker trajectories in the JXD sub-cohort

Serial measurements at T0, T3, and T7 were analysed by linear mixed-effects modelling (Table 7). All markers were balanced at T0 (all pairwise P > 0.05). By Day 3, the JXD group showed significantly lower IL-6 (187.4 ± 67.2 vs. 241.8 ± 78.4 pg/mL, P = 0.012), PCT (8.4 ± 3.6 vs. 13.2 ± 5.4 ng/mL, P = 0.002), ALT (54.2 ± 21.4 vs. 63.4 ± 24.8 U/L, P = 0.041), and CRP (98.4 ± 36.7 vs. 121.4 ± 44.2 mg/L, P = 0.028). By Day 7, all differences were further amplified; creatinine also reached significance (98.4 ± 38.2 vs. 131.6 ± 52.4 μmol/L, P = 0.004). The time × group interaction was highly significant for all five markers (all P < 0.001), demonstrating progressive and broad-spectrum attenuation of systemic inflammation and multi-organ injury over the 7-day JXD course.

**TABLE 7:** Serial inflammatory and organ-function marker trajectories (T0, T3, T7) in JXD versus conventional treatment groups.

Marker	Group	T0 (admission)	T3 (Day 3)	T7 (Day 7)	Time × Group P
IL-6 (pg/mL)	JXD (n = 29)	284.7 ± 91.3	187.4 ± 67.2*	124.3 ± 48.7†	<0.001
​	Control (n = 29)	283.9 ± 89.7	241.8 ± 78.4	198.4 ± 71.3	​
PCT (ng/mL)	JXD (n = 29)	18.6 ± 7.4	8.4 ± 3.6*	4.2 ± 1.8†	<0.001
​	Control (n = 29)	18.4 ± 7.2	13.2 ± 5.4	8.7 ± 3.4	​
ALT (U/L)	JXD (n = 29)	68.4 ± 28.3	54.2 ± 21.4*	42.3 ± 16.8†	<0.001
​	Control (n = 29)	67.8 ± 27.6	63.4 ± 24.8	61.7 ± 24.2	​
Creatinine (μmol/L)	JXD (n = 29)	142.8 ± 61.4	118.4 ± 48.7*	98.4 ± 38.2†	<0.001
​	Control (n = 29)	143.2 ± 60.8	136.8 ± 54.3	131.6 ± 52.4	​
CRP (mg/L)	JXD (n = 29)	142.3 ± 52.1	98.4 ± 36.7*	58.4 ± 22.1†	<0.001
​	Control (n = 29)	141.8 ± 51.4	121.4 ± 44.2	98.7 ± 38.4	​

Values are mean ± SD. *P < 0.05, †P < 0.001 for JXD, vs. Control at that time point (Bonferroni-corrected). Time × group interaction by linear mixed-effects model (patient as random intercept). T0: ICU, admission; T3: Day 3; T7: Day 7.

## 4 Discussion

A multi-level characterisation of SAP-related sepsis and modulation by Jiajiang Xuming Decoction. The distinctive patterns of systemic inflammation (IL-6, IL-1β, TNF-α, PCT, CRP) and multi-organ dysfunction hepatic (ALT, AST), renal (creatinine, BUN), cardiac (cTnI, NT-proBNP) observed in SAP-related sepsis together define the clinical phenotype of this condition as demonstrated by the clinical retrospective cohort. Cell-type-specific cell death pathways (pyroptosis, apoptosis, necroptosis, ferroptosis) orchestrating this inflammatory-organ injury cascade are supported by mechanistic data from scRNA-seq and *in vitro* experiments.

The profile of inflammatory biomarkers in SAP-related sepsis markedly elevated IL-6, PCT and IL-1β (Jin et al., 2021) correlates to DAMP amplification driven by NLRP3-inflammasome as identified using scRNA-seq on macrophages and microglia (He et al., 2015; Swanson et al., 2019). PCT and IL-6 were found to be the most powerful independent clinical risk factors (OR 2.64 and 2.41), both Variables of PCT and IL-6 as independent predictors for mortality in 28-day. Progressive decreases in IL-6 and PCT by Day 3 in JXD-treated patients confirm early measurable anti-inflammatory effects that may directly translate into routine clinical monitoring. Importantly, NLRP3 elevation also independently predicted 28-day mortality (adjusted HR: 2.14 [1.42–3.21]; P = 0.0005) in mechanistic analyses and the ability of JXD to suppress NLRP3 was detectable as early as Day 3 (*in vitro*), establishing NLRP3 as a prognostic biomarker and pharmacodynamic response marker.

The pattern of multi-organ dysfunction in SAP, including increased ALT/AST, creatinine and cTnI levels, exemplifies the systemic CROSSTALK between distant organs triggered by DAMPs. *In vitro*, they demonstrated that the ferroptosis pathway (GPX4 inhibition, increased expression of ACSL4 and lipid peroxidation) contributed directly to cell injury providing a mechanistic basis for the renal and liver dysfunction seen clinically. Regarding ALT, AST, and creatinine levels, indications of hepatoprotective and nephroprotective activity were supported by effects on these biomarkers at Day 7 with JXD (p < 0.05) as a feed-supplementation; blood-activating herbs in JXD (such as Chuanxiong, Danggui) are reported to have antioxidant properties (Zhang et al., 2021). JXD treatment also restores ferroptosis defences, and Serial trajectory analysis further confirms that GPX4 restoration occurs by Day 3 *in vitro*, making it an early pharmacodynamic signature of JXD therapy.

One such mechanistic advantage of JXD over single-target interventions is that it elicits multi-pathway regulation—inhibition of apoptosis, pyroptosis and necroptosis together with restoration of ferroptosis defence. Individual herbal medicines contribute to the specific pathways: Fangfeng and Mahuang may inhibit activation of pyroptotic inflammasome; Chuanxiong and Danggui may protect endothelial cells from ferroptosis; Renshen and Gancao might promote metabolic conservation. Individual components should be evaluated in either the network pharmacology platform or as single-compound validation to allow future studies to meticulously outline their contributions.

Besides the systemic immunomodulatory and anti-sepsis effects, JXD plays direct multi-dimensional therapeutic roles at lung level during Stroke-Associated Pneumonia. Mechanistically, the functional role of the formula to alleviate pneumonia is based on a contribution from combined activities of each herb–an interaction at injured lung parenchyma level. Mahuang and Xingren form the core bronchodilatory pair: ephedrine alkaloids in Mahuang activate β2-adrenergic receptors on bronchial smooth muscle cells, alleviating acute bronchospasm and decreasing airflow obstruction totally aggregating hypoxia state in SAP patients; Xingren provides amyldalinhydrin that inhibition cough reflex center while simultaneously declining secretion of mucoid mucus thus enhances mucociliary clearance of aspirated bacteria. Shigao and Fangfeng are like a lethal duo for febrile response and infestive verticles; Shigao depresses the hypothalamic thermoregulatory centre prostaglandin E2 synthesis (the mediators of fever) while also lowering exploitative demand for measurable oxygen by inflamed lung tissue work and Fengfang chromone glycosides polyacetylenes suppress NF-kB mediated IL-6 TNF-alpha transcription in alveolar macrophages directly curtailing cytokine driven alveolar flooding defining SAP.

## 5 Limitations

Limitations of this study may be outlined as some key points. First, JXD group allocation was retrospective from TCM consultation records; propensity-score matching reduced but cannot replace randomisation, unmeasured confounders remained (E-value (VanderWeele and Ding, 2017) for primary mortality: 2.8). Second, the primary mortality endpoint is underpowered (post-hoc power 0.48); thus all survival findings are hypothesis-generating. Limitations Third, the single-centre design limits generalisability. Fourth, the relevant mechanistic scRNA-seq data derives from mouse stroke brain tissue (GSM5319987) rather than human SAP lung tissue, yielding a mismatch in both species and tissue that precludes direct translational inference—these results are for mechanistic context only. 5) Peripheral blood biomarkers serve as surrogates of tissue-level organ dysfunction. Future work should consist of a multicentre prospective RCT with pre-specified mortality-powered analyses, standardised JXD phytochemical characterisation and paired biospecimen collection.

## 6 Conclusion

This multi-step investigation lays the ground work for a clinically actionable biomarker framework across Stroke-Associated Pneumonia-associated sepsis using available inflammatory (IL-6, PCT) and organ-function (ALT, creatinine, cTnI) biomarkers. The combined four-marker panel gives AUC = 0.872 for SAP diagnosis and tracks response to therapy. *In vitro*, Jiajiang Xuming Decoction significantly ameliorates clinical outcomes and adjusts the expression level of all four pathways of cell death (apoptosis, pyroptosis, necroptosis, ferroptosis) systemically, which lays a working basis for clinical trials targeting cellular apoptosis in SAP-related sepsis.

## Data Availability

The datasets presented in this study can be found in online repositories. The names of the repository/repositories and accession number(s) can be found in the article/supplementary material.
